# Cellular Senescence and Iron Dyshomeostasis in Alzheimer’s Disease

**DOI:** 10.3390/ph12020093

**Published:** 2019-06-19

**Authors:** Shashank Masaldan, Abdel Ali Belaidi, Scott Ayton, Ashley I. Bush

**Affiliations:** Melbourne Dementia Research Centre, The Florey Institute of Neuroscience and Mental Health, The University of Melbourne, Parkville, VIC 3052, Australia; shashank.masaldan@florey.edu.au (S.M.); abdel.belaidi@florey.edu.au (A.A.B.); scott.ayton@florey.edu.au (S.A.)

**Keywords:** Alzheimer’s disease, iron homeostasis, ferroptosis, senescence, chelators

## Abstract

Iron dyshomeostasis is a feature of Alzheimer’s disease (AD). The impact of iron on AD is attributed to its interactions with the central proteins of AD pathology (amyloid precursor protein and tau) and/or through the iron-mediated generation of prooxidant molecules (e.g., hydroxyl radicals). However, the source of iron accumulation in pathologically relevant regions of the brain and its contribution to AD remains unclear. One likely contributor to iron accumulation is the age-associated increase in tissue-resident senescent cells that drive inflammation and contribute to various pathologies associated with advanced age. Iron accumulation predisposes ageing tissue to oxidative stress that can lead to cellular dysfunction and to iron-dependent cell death modalities (e.g., ferroptosis). Further, elevated brain iron is associated with the progression of AD and cognitive decline. Elevated brain iron presents a feature of AD that may be modified pharmacologically to mitigate the effects of age/senescence-associated iron dyshomeostasis and improve disease outcome.

## 1. Introduction

Alzheimer’s disease (AD) is the most common type of dementia. Pathological hallmarks of AD are the accumulation of extracellular amyloid plaques seeded by aggregated amyloid beta peptide (Aβ) and intracellular neurofibrillary tangles (NFTs) composed of hyper-phosphorylated microtubule-associated protein tau. The accumulation of Aβ is considered a toxic component of pathology and has been a primary target of clinical strategies [1]. However, strategies that have focused on reducing Aβ burden, including those that have demonstrated the lowering of plaque burden to normal levels, have not been successful in slowing cognitive decline in AD patients [1,2,3]. A careful re-examination of the factors that may lead to AD and contribute to cognitive decline may facilitate the formulation of new therapeutic strategies to prevent or arrest disease processes. Homeostatic regulation of iron is one such pathway amenable to therapeutic targeting, and it has been observed to be perturbed in several neurodegenerative disorders in addition to AD [4].

Iron is essential for life processes and cellular functions. These include essential “housekeeping” functions such as cellular respiration, DNA synthesis, and cell division, as well as specialized cellular functions such as oxygen transport and neurotransmission [4,5,6]. The ability of iron to cycle through its oxidation states is fundamental to its biological utility but can lead to oxidative damage of biomolecules resulting in cellular dysfunction [4,5,6]. This has led to the evolution of tightly regulated homeostatic mechanisms to ensure iron availability and mitigate toxicity [4,5,6]. However, the brain accumulates iron with age and several neurodegenerative conditions are associated with increased iron levels in affected regions of the brain [4,5].

**Figure 1:**
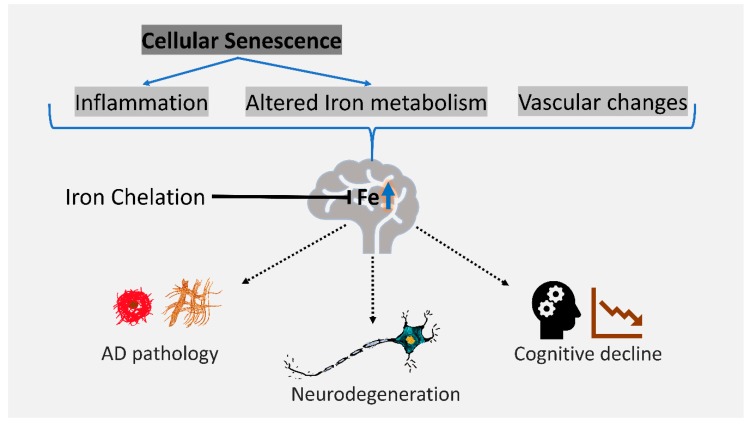
Cellular senescence is a potential contributor to the age-associated accumulation of brain iron. Factors that influence brain iron with age include inflammation, altered vasculature, and altered metabolism. Elevated brain iron is associated with Alzheimer’s disease (AD) pathology, cognitive decline, and may lead to neuron loss via iron-dependent oxidative cell death such as ferroptosis. Iron chelation may mitigate some of these effects and alleviate AD progression.

The cause of age-associated iron accumulation in brain regions relevant to AD and its impact on disease are relevant questions to determine the utility of brain iron redistribution as a therapeutic strategy for AD. In this review we explore the contribution of iron to AD and describe the potential contribution of the proinflammatory senescence program to brain iron accumulation (Figure 1). Notably, while iron accumulation in AD may not be sufficiently high to result in iron toxicity [6], iron dyshomeostasis and elevated iron predisposes and enhances the susceptibility of brain tissue to oxidative dysfunction (e.g., lower glutathione, increased lipid peroxidation, and increased reactive oxygen species) and accelerates cell death modalities such as ferroptosis (reviewed in [4]). Finally, we describe the therapeutic opportunities that may be explored to alleviate AD through pharmacological chelation of iron in the brain.

## 2. Iron Dyshomeostasis is Associated with AD

In the brain, iron accumulation is observed in regions affected by AD such as the parietal cortex, motor cortex, and hippocampus [7,8,9,10,11,12,13,14,15]. The intensity of iron accumulation, observed by histology, in the frontal cortex is different between the subtypes of AD. This can be used to distinguish between sporadic (late onset) and familial (early onset) AD [16] and reflects disease severity [17,18]. Overall, patients with familial AD are affected more in magnetic resonance imaging (MRI) scores compared to patients with sporadic AD, which may reflect higher iron accumulation in familial AD [16]. Further, the forms of iron observed in AD patients vary in their magnetic moment (measured via superconducting quantum interference device magnetometry) when compared with those from age- and gender-matched controls [19]. Magnetic moment is the property of a substance that determines the torque it experiences when influenced by an external magnetic field. Thus, change in magnetic moment reflects a change in the molecular state of iron and indicates possible dysregulation of iron homeostasis [19]. For example, a higher magnetic moment in AD brain tissue vs. control tissue in the absence of changes in the concentration of magnetite (an oxide of iron that can be magnetized) may indicate the larger size of magnetite particles in AD brains. This in turn suggests a dysfunction of iron storage in ferritin (iron storage protein) and/or accumulation of Aβ in AD tissue [19]. Further, iron levels measured post-mortem are elevated in the inferior temporal cortex only in patients diagnosed with AD during their lives, with AD pathology confirmed post-mortem [20].

Iron is associated with the pathological lesions of AD [17,21,22,23,24,25,26,27,28,29]. Some studies implicate iron as a direct contributor to AD pathology by promoting the aggregation and oligomerization of Aβ peptides [17,26,28,29,30,31,32,33]. Levels of iron and ferritin (iron storage protein) in brain tissue are associated with the amount of amyloid deposition [17,34,35]. Iron is accumulated in amyloid plaques as a mineralized magnetite species in mouse and human models of amyloid deposition [26,27,28,29]. Aβ may enhance iron mineralization as Aβ peptides can lead to the production of iron-mineral nanoparticles in vitro [36]. It is hypothesized that the oxidative damage associated with the aggregation of Aβ is due to redox active metals (e.g., iron and copper) to which it binds, leading to the production of oxidants such as hydrogen peroxide [37,38].

Iron may impact the production of Aβ by enhancing the translation and amyloidogenic processing of amyloid precursor protein (APP) [39,40,41,42,43,44,45]. APP, when processed through a non-amyloidogenic pathway, is cleaved by α-secretase followed by cleavage by γ-secretase. Iron availability may perturb this process through the aberrant binding of iron response proteins to putative iron response elements on APP mRNA [43,44,45]. Additionally, iron can mediate tau phosphorylation and aggregation [46,47,48]. These events can be mitigated by the chelation of iron [49]. Tau accumulation in NFTs is associated with an induction of heme oxygenase-1 which can exacerbate oxidative stress through the release of iron by the breakdown of heme [45,50,51,52].

Brain iron and ferritin are associated with cognitive loss in AD. Iron was strongly associated with the rate of cognitive decline 12 years prior to death in the subjects from the Memory and Ageing Project (*n* = 209) [20]. While the direct measurement of brain iron is challenging, ferritin levels in cerebrospinal fluid can be used as a proximate reporter of brain iron load. While brain iron levels are reflected in CSF (cerebrospinal fluid) ferritin, they may also be impacted by the inflammation status of the brain. Regardless, CSF ferritin can predict cognitive decline and the transition from mild cognitive impairment to AD [53]. Ferritin in the CSF predicts the rate of decline in brain metabolism (proximate indicator of neurodegeneration), as measured by fluorodeoxyglucose positron emission tomography (FDG-PET) in subjects with high amyloid pathology (high CSF t-tau/Aβ42 ratio) but not in subjects with low amyloid pathology [54]. Another longitudinal study conducted over six years determined that elevated magnetic susceptibility in the hippocampus, determined by an MRI technique called quantitative susceptibility mapping (QSM), is a strong predictor for an accelerated rate of cognitive decline in amyloid positive subjects [55]. QSM may also be used to longitudinally and non-invasively monitor amyloid accumulation and iron deposition, and may serve as a diagnostic aid for AD [35]. Taken together, these studies suggest that iron is important for cognitive deterioration when there is underlying pathology.

## 3. Cellular Senescence is Associated with AD and Iron Dyshomeostasis

Cellular senescence is a proinflammatory cell fate associated with several age-related disorders, including AD [56,57,58]. The senescence phenotype is classically described in cultured cells undergoing terminal replicative arrest [59] that display enlarged cell morphology and characteristic alterations of their chromatin, secretory profile (senescence-associated secretory phenotype (SASP)), and cell cycle regulatory proteins (cyclins and cyclin-dependent kinases) [60]. Senescence in culture is typically induced through sub-culturing to replicative exhaustion, inducing DNA damage (through oxidative stress, ionizing radiation, or pharmacological agents), or aberrant oncogenic activation (e.g., overexpression of H*ras*^V12^) [60]. The occurrence of senescent cells in tissue is now widely accepted [61,62,63,64,65] and several functions have been ascribed to them, including involvement in wound healing [66,67], tissue repair [68], and embryonic development (developmental senescence) [69,70]. Despite these beneficial roles, the persistent accumulation of senescence in tissue with age is associated with age-associated pathologies and functional decline. The clearance of senescent cells from tissue in mice can alleviate pathologies related to ageing [56,57,58,71,72,73,74,75,76,77,78,79,80,81]. While the overall burden of senescent cells in ageing tissue is low, these cells persistently propagate degenerative and proinflammatory conditions in their microenvironment [56,57,58,71,72,73,74,75,76,77,78,79,80,81,82,83]. In the brain the senescence program can be triggered in astrocytes and microglia, with recent evidence suggesting that even neurons may display a senescent signature despite being post-mitotic. A more flexible definition of senescence based on the outcomes of senescent cell phenotypes (e.g., chronic inflammation) may be relevant to explain age-related pathologies in vivo. As the molecular changes associated with normal ageing that promote AD are yet to be fully defined, the impact of cellular senescence on age-related neuroinflammation, decline in cognition, and AD is currently unknown [75].

Evidence for the induction of senescence in cells of the brain and its links with neurodegenerative disorders is steadily increasing. In cell culture, astrocytes exposed to hydrogen peroxide (oxidative stress) or irradiation display markers of senescence, such as senescence-associated βgalactosidase staining (SAβgal) (visualized by enzymatic activity assay for lysosomal βgalactosidase at pH 6.0) and elevated expression levels of p16^INK4A^ and p21 (measured via quantitative PCR for the increase in transcript levels or Western blotting to reflect changes in protein abundance), and develop a proinflammatory secretory phenotype similar to the SASP observed in senescent fibroblasts [84,85,86]. Similarly, irradiated neuronal cells show features of senescence (SAβgal positivity) and become susceptible to the senolytic pharmacological cocktail of dasatinib (kinase inhibitor anti-cancer drug) and quercetin (plant-derived flavonoid) [58]. In human brain tissue an increase in the burden of p16^INK4A^-expressing astrocytes is observed with age [87]. Cellular senescence in neuronal progenitor cells is implicated in the reduced remyelination observed in progressive multiple sclerosis [88]. In mouse models, senescence in the brain has been observed in response to physiological (e.g., obesity-induced [89]) and physical (e.g., traumatic brain injury following controlled cortical impact [90]) stressors.

In the context of AD, astrocytes expressing p16^INK4A^ are enriched in the frontal cortex of AD patients compared to age-matched non-AD adults [87]. Further, oligodendrocyte progenitor cells displaying a senescent phenotype (high p21 expression) are associated with amyloid plaques in the brains of human AD patients [58]. Recently, studies using mouse models that overexpress mutant tau and phenocopy aspects of AD have indicated a strong link between the induction of cellular senescence and the appearance of AD pathology [56,57]. In particular, the ablation of senescent cells from the tissue of these mice led to a reduction in the phosphorylation and aggregation of tau [56,57]. In another mouse model of AD (APP/PS1), senolytic treatment, using a combination of dasatinib and quercetin that reduced the burden of senescent cells associated with amyloid plaque, lowered Aβ load, reduced neuroinflammation, and reduced cognitive defects [58]. Interestingly, Aβ can induce senescence in cultured oligodendrocyte progenitor cells [58] and drive SASP in cultured epithelial cells and fibroblasts via CD36 [91]. This may indicate that AD pathologies can further sustain/enhance tissue-resident senescence burden. Taken together, these studies suggest that senescence induction occurs in the brain and is associated with AD.

While the clearance of senescent cells in tissues of mice has now been demonstrated to mitigate age-associated pathologies, including AD, the impact of removing them in tissues of longer-living mammals, including humans, is yet to be determined. A small (*n* = 14) first in-human clinical trial of the senolytic cocktail of dasatinib and quercetin in idiopathic pulmonary fibrosis (IPF), a fatal cellular senescence-associated disease [76], provided no conclusive evidence of senescence clearance or reduction in SASP despite achieving its primary endpoints (retention and completion rate; both 100%) and some benefit in the physical function of patients [92]. Considering the known functional benefits of senescence in wound healing and tissue repair, as well as other potential benefits that may be currently unknown, the life-long clearance of senescent cells in humans may be deleterious. Further, therapeutic strategies based on the complete clearance of senescent cells may be difficult to administer in disorders like AD where the disease develops over several decades. An alternative strategy is to target specific phenotypic features of senescent cells that are relevant in certain disease settings. Iron accumulation is one such feature that is observed in senescent cells and is of relevance in AD [82].

Senescent cells in vitro display aberrant iron homeostasis [82], and there are some indications that their abundance influences iron levels in ageing tissue [82,93,94]. Senescent cells display elevated iron and a concomitant increase in ferritin and markers of oxidative stress in vitro [82,93,94,95]. Iron is known to promote the induction of senescence in cultured microglia [96]. Iron chelators such as deferoxamine and deferiprone can reduce and prevent the accumulation of iron and ferritin observed in cellular senescence in vitro [82]. The chelation of iron in *Caenorhabditis elegans*, a model organism of ageing that displays iron dysfunction in senescent intestinal cells [97], leads to a reduction in iron-dependent oxidation and cell death [98]. In the context of brain tissue, SASP may drive ferritin expression in neurons and glia as an acute phase response which may enhance their susceptibility to the iron-mediated cell death process, ferroptosis. The effects of iron chelation on senescence-associated iron accumulation and its impact on SASP or ferroptotic vulnerability are yet to be explored in vivo and present a therapeutic opportunity to treat AD (Figure 1).

## 4. Iron as a Therapeutic Target in AD

Iron neurochemistry as a modifiable feature to treat AD has generated renewed interest since clinical trials for iron chelators have shown promise recently in other neurodegenerative disorders such as Parkinson’s disease (PD) and motor neuron disease (MND) [6,99,100,101,102,103,104]. The efforts towards the “iron hypothesis” of AD have also been bolstered by the unravelling of complex molecular crosstalk between iron regulatory proteins and the suspected players of AD pathology.

Historically, the first study that explored iron chelation against AD was published in 1991, which tested the effectiveness of intramuscular application of the iron chelator deferoxamine in 48 patients over two years [105]. The study showed that low-dose administration of this iron chelator slowed the clinical progression of dementia associated with AD compared with controls. A decade later, a pilot phase 2 clinical trial in patients with moderately severe AD using clioquinol (PBT1), a drug inhibiting zinc and copper ions from binding to Aβ, was conducted on 36 patients [106]. A positive clinical effect, corresponding to a reduction in the rate of cognitive decline, was seen in the more severely affected patients. Moreover, a biological effect corresponding to a decline in plasma Aβ42 levels was observed. Currently, the safety and efficacy of deferiprone, an iron chelator that passes the blood–brain barrier, is under evaluation in a phase 2 randomized placebo-controlled clinical trial in participants with prodromal AD and mild AD (NCT03234686).

## 5. Conclusions

Despite the ever-increasing socioeconomic burden of AD, there is frustratingly no disease-modifying treatment for this affliction. The historic and emerging evidence that iron contributes to the clinical progression of AD should not be ignored as a potential avenue for therapy development. The reported benefits for iron chelation in other neurodegenerative diseases such as PD and MND should open the possibility of pharmacological manipulation of brain iron as an alternative therapeutic approach for AD. The perturbed iron homeostasis observed in senescent cells presents a possible cell-specific target of iron chelation therapy which may enhance the efficacy of approaches aimed at lowering age/pathology-related iron accumulation. Further, the strategy to modify specific phenotypic features (e.g., reducing iron accumulation using chelators such as deferiprone) of senescent cells that contribute to pathology may be of benefit in the human context where the complete ablation of senescent cells from tissue may be difficult or even deleterious due to a loss of beneficial effects associated with senescence (e.g., tissue repair and wound healing). The consequence of cellular senescence on the iron homeostasis of the brain requires further characterization. This may open additional avenues for the development of new classes of drugs that may provide benefits for AD patients and provide strategies for halting their decline and delaying the onset of neurodegeneration.
